# Dealing with staff diversity in German hospitals: A comparative analysis of doctors and nurses: Lisa Peppler

**DOI:** 10.1093/eurpub/ckac130.147

**Published:** 2022-10-25

**Authors:** L Peppler, K Molzberger, P Beck, D Matusiewicz, L Schenk

**Affiliations:** 1 Institute of Medical Sociology and Rehabilitation Science, Charité University Medicine, Berlin, Germany; 2 Center for Diversity Research in Teaching, Universität Bonn, Bonn, Germany; 3 Institute for Health & Social Sciences, FOM University of Applied Sciences gGmbH, Essen, Germany

## Abstract

**Background:**

Germany is increasingly recruiting foreign healthcare staff due to the shortage of skilled workers. This diversity of professional and cultural backgrounds poses a challenge to everyday life in inpatient care. Previous studies have focused on the renegotiation of professional identities and competencies in nursing or medicine. In contrast, this study sheds light on group-specific mechanisms through a comparative analysis: How do doctors and nurses deal with diversity in the workplace? Where do profession-specific differences emerge and what does this mean for future interventions?

**Methods:**

Eight group discussions (June 2019 to October 2020) were conducted with groups of doctors and nurses with and without a migration background in four hospitals in two federal states in Germany; including 22 nurses and 10 doctors (n = 32). The data were analysed using the documentary method to examine professional meaning-making processes. The results were validated intersubjectively.

**Results:**

The respective handling of diversity in the workplace is influenced by different professional group identities. The situation is precarious for nurses with a migration background - especially for those with an academic degree, as nursing is still an apprenticeship profession in Germany. In the medical profession, on the other hand, diversity does not lead to significant controversies, even if cultural differences are discussed.

**Conclusions:**

Dealing with diversity is negotiated within professional groups. As nursing or medical ‘communities of practice’ (E. Wenger), these have a mediating role through which they can mitigate institutional and individual barriers to the integration of migrants in the workplace.

**Key messages:**

- Physicians and nurses need more specific, iterative cross-occupational and cross-cultural education.
- Management should support in accommodating different expectations and abilities.

